# Novel Case Report of a Rare Symptomatic Orofacial Burkitt’s Lymphoma

**DOI:** 10.1155/crid/9669783

**Published:** 2026-04-29

**Authors:** Mona Shameli, Pedram Hajibagheri, Amirreza Hendi, Mahsa Koochaki

**Affiliations:** ^1^ Student Research Committee, School of Dentistry, Guilan University of Medical Sciences, Rasht, Iran, gums.ac.ir; ^2^ Dental Sciences Research Center, Department of Oral and Maxillofacial Medicine, School of Dentistry, Guilan University of Medical Sciences, Rasht, Iran, gums.ac.ir; ^3^ Dental Sciences Research Center, Department of Prosthodontics, School of Dentistry, Guilan University of Medical Sciences, Rasht, Iran, gums.ac.ir

**Keywords:** Burkitt lymphoma, case report, head and neck neoplasms, mandibular neoplasms, toothache

## Abstract

Burkitt lymphoma is a very fast‐growing non‐Hodgkin lymphoma (NHL) characterized by rapid tumor progression and increased lymphocyte proliferation. It often involves the jaw and facial bones. The current case report represented a 37‐year‐old male patient with Burkitt’s lymphoma who sought dental care for persistent and dull pain and paresthesia on the left side of his mandible extending to the periauricular region. The initial diagnosis suggested a dental origin, leading to the lower left third molar (LL8) extraction. Despite this, the patient continued to experience worsening pain, which radiated to the lower extremities, accompanied by fatigue and weakness. The patient was referred to an oral and maxillofacial medicine specialist, where ultrasonography (US) revealed hypoechoic masses. A subsequent biopsy of a submandibular mass confirmed the diagnosis of Burkitt’s lymphoma. The patient was referred to an oncologist and underwent chemotherapy and radiotherapy. After 3 years of follow‐up, the patient is in remission. This unique case presentation related to systemic involvement as well as generalized bone pain (suggesting systemic involvement or more advanced disease) alone underscores the importance of a thorough diagnostic approach when facing unusual or persistent symptoms. It can also significantly impact future patient care by highlighting the need for a comprehensive evaluation and the importance of maintaining a high level of suspicion, particularly in cases where the initial diagnosis does not fully explain the patient’s symptoms.

## 1. Introduction

Burkitt’s lymphoma is a highly aggressive type of non‐Hodgkin lymphoma (NHL) characterized by rapid tumor growth and increased lymphocyte proliferation, often involving the abdomen, jaw, and facial bones [1]. Burkitt’s lymphoma is classified into three main subtypes: endemic, sporadic, and immunodeficiency‐associated. Endemic Burkitt’s lymphoma primarily affects children in sub‐Saharan Africa, while the sporadic form occurs globally. The immunodeficiency‐associated subtype is most commonly seen in individuals with HIV, though it can also affect patients who have received organ transplants [2, 3] and individuals with hereditary immunodeficiency. The sporadic form of Burkitt’s lymphoma has an estimated annual incidence of 2.5 cases per 1 million adults [4]. Although it is a rare condition, early diagnosis and immediate treatment are crucial due to its rapid progression because it is a highly chemotherapy‐sensitive disease, and treatment is achieved with chemotherapy alone [5]. Burkitt lymphoma is characterized by chromosomal translocations, primarily involving the MYC oncogene. The most common translocation occurs between MYC and an immunoglobulin locus, resulting in its constitutive expression, a defining feature observed in all cases [6].

The clinical presentation of Burkitt’s lymphoma varies depending on the subtype and other factors. In endemic Burkitt’s lymphoma, the disease commonly presents with jaw and facial complications, and may also involve the ileum, cecum, gonads, kidneys, and breasts. In contrast, sporadic Burkitt’s lymphoma typically affects the ileocecal region, with less frequent jaw involvement. Immunodeficiency‐associated Burkitt’s lymphoma often impacts the ileum, cecum, lymph nodes, and bone marrow. Central nervous system (CNS) involvement can also occur in all disease variants. Imaging techniques are valuable for detecting head and neck involvement in Burkitt’s lymphoma. Ultrasonography (US) helps identify enlarged lymph nodes in the neck. At the same time, computed tomography (CT) and magnetic resonance imaging (MRI) are effective in detecting extranodal involvement and bone destruction, as well as staging the disease [7]. Patients are categorized according to the National Cancer Institute (NCI) staging system. This system defines following stages: Stage A: Single extra‐abdominal site. Stage AR: An intra‐abdominal tumor for which greater than 90% was surgically resected. Stage B: Multiple extra‐abdominal sites. Stage C: Intra‐abdominal tumor. Stage D: Intra‐abdominal tumor with single or multiple extra‐abdominal sites [8].

Laboratory blood test and immunohistochemistry (IHC) assessment can facilitate the diagnosis. IHC is a technique that uses antibodies to detect specific proteins in a tissue sample. In the case of Burkitt’s Lymphoma, IHC can help identify the presence of certain markers, such as CD10, BCL6, and BCL2, which are characteristic of this disease. Oral signs of BL can include the molar teeth loosening, primary molars falling out earlier than permanent molars in children, the gums becoming more prominent, and swelling in the areas around the teeth and jaws. On X‐rays, BL can be seen as osteolytic lesions around the permanent tooth buds. Some of the X‐ray signs can be present even if there are no obvious clinically detectable tumors in the jaw [9]. Extracting impacted third molars is a routine procedure in oral and maxillofacial surgery. This present case illustrates the diagnostic journey of a young patient initially seeking treatment for persistent jaw pain. The pain was initially attributed to a dental issue, leading to the extraction of the lower left third molar (LL8), until further evaluation by specialists revealed the underlying condition as Burkitt’s lymphoma.

Patients diagnosed with head and neck extranodal NHL often respond positively to treatment with immuno‐polychemotherapy. However, certain factors can indicate a poor prognosis. These include low hemoglobin levels, higher levels of β2‐microglobulin and lactate dehydrogenase (LDH). Understanding these factors can help healthcare providers tailor treatment plans and offer more personalized care [10].

In this article, we describe the diagnosis and treatment outcome of a patient with an unusual presentation of Burkitt lymphoma of the jaw, which mimicked dental pain yet lacked other distinguishing clinical and radiographic features and did not fit neatly into any of the three standard WHO–defined variants (endemic, sporadic, or immunodeficiency‐associated).

## 2. Case Presentation

A 37‐year‐old male visited his private dentist complaining of dull pain and paresthesia on the left side of his mandible, extending to the periauricular region for the past 3 days. These symptoms, particularly the paresthesia (tingling or numbness), can be indicative of nerve involvement, which is a less common but important manifestation of Burkitt’s Lymphoma. An orthopantomogram (OPG) was performed, and no radiographic abnormalities or significant caries were observed in the third molar during the initial examination. With no other identifiable cause, the pain was attributed to a dental origin, leading to the extraction of the third molar. However, the pain persisted and worsened, prompting the prescription of antibiotics to rule out infection. Following oral antibiotic therapy, a parenteral regimen consisting of six doses of penicillin‐G over 1 week was administered, yet the pain continued to intensify.

The discomfort began to radiate from the jaw to the lower extremities, particularly affecting the knees and other joints, causing severe pain. The patient also experienced worsening lethargy, which became debilitating. Finally, 30 days after symptom onset, the patient sought further evaluation from an oral and maxillofacial medicine specialist. Upon examination, palpable lymph nodes were detected in both the submandibular and jugulodigastric regions, categorizing the patient in Stage B of the disease. Suspecting inflamed lymph nodes or potential malignancy, the surgeon recommended soft tissue US of the neck for further investigation. US is a noninvasive imaging technique that uses high‐frequency sound waves to produce images of the inside of the body. In the case of Burkitt’s Lymphoma, US can help identify enlarged lymph nodes and hypoechoic masses, which are indicative of the disease.

**Figure 1:**
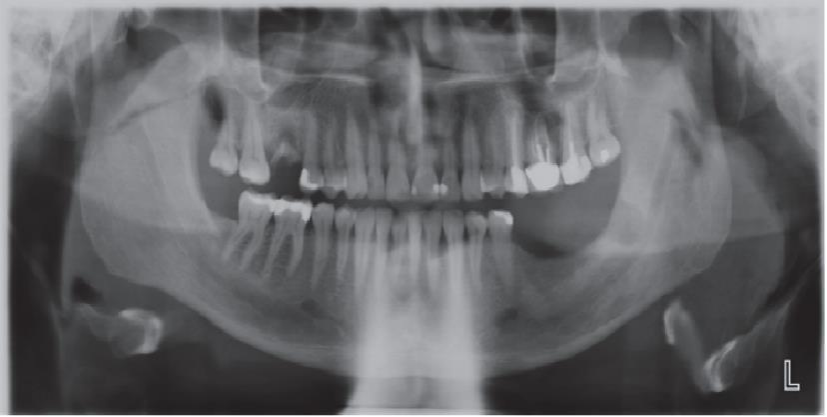
This is the postextraction orthopantomogram (OPG). A patch of radiolucency inferior to LL7/8 was seen.

A diagnostic ultrasound of the neck revealed two hypoechoic masses. The first was a lobulated hypoechoic mass measuring 26 mm × 26 mm. In comparison, the second was an ovoid hypoechoic mass measuring 22 mm × 14 mm, positioned anteriorly to the left carotid artery and jugular vein at the upper level 2. Additionally, a heterogeneous ovoid mass measuring approximately 50 mm × 25 mm was identified in the left submandibular region. A follow‐up OPG was also performed after the extraction of the LL7, revealing a radiolucent area located inferior to the LL7 and LL8 regions (Figure 1).

**Figure 2:**
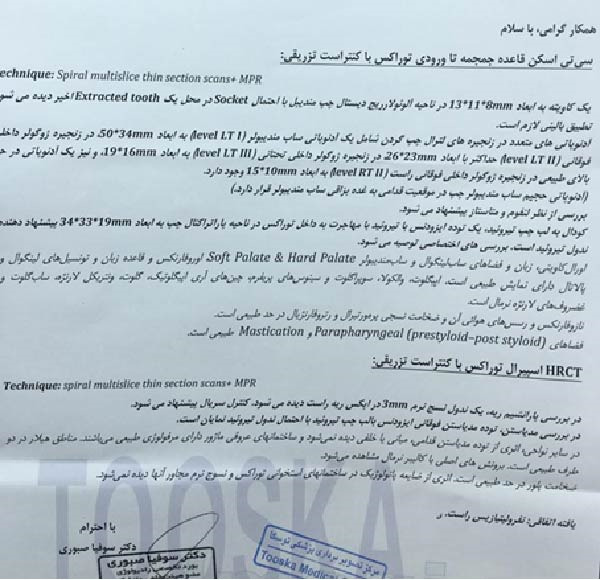
Contrast‐enhanced CT scan from the skull base to the thoracic inlet.

The patient was referred for further evaluation with a contrast‐enhanced CT scan from the skull base to the thoracic inlet. The scan revealed a cavity measuring 13 mm × 11 mm × 8 mm in the distal left mandibular alveolar ridge, most likely a residual socket from a previous tooth extraction. Multiple lymph nodes were identified in the left lateral cervical chains, including a left submandibular lymph node (Level LT I) measuring 50 mm × 34 mm, a node in the left upper internal jugular chain (Level LT II) measuring up to 26 mm × 23 mm, and a node in the left lower internal jugular chain (Level LT III) measuring 19 mm × 16 mm. Additionally, there was a lymph node in the superior aspect of the right upper internal jugular chain (Level RT II) measuring 15 mm × 10 mm, as well as a bulky left submandibular lymph node situated anteriorly adjacent to the submandibular salivary gland (Figure 2).

**Figure 3:**
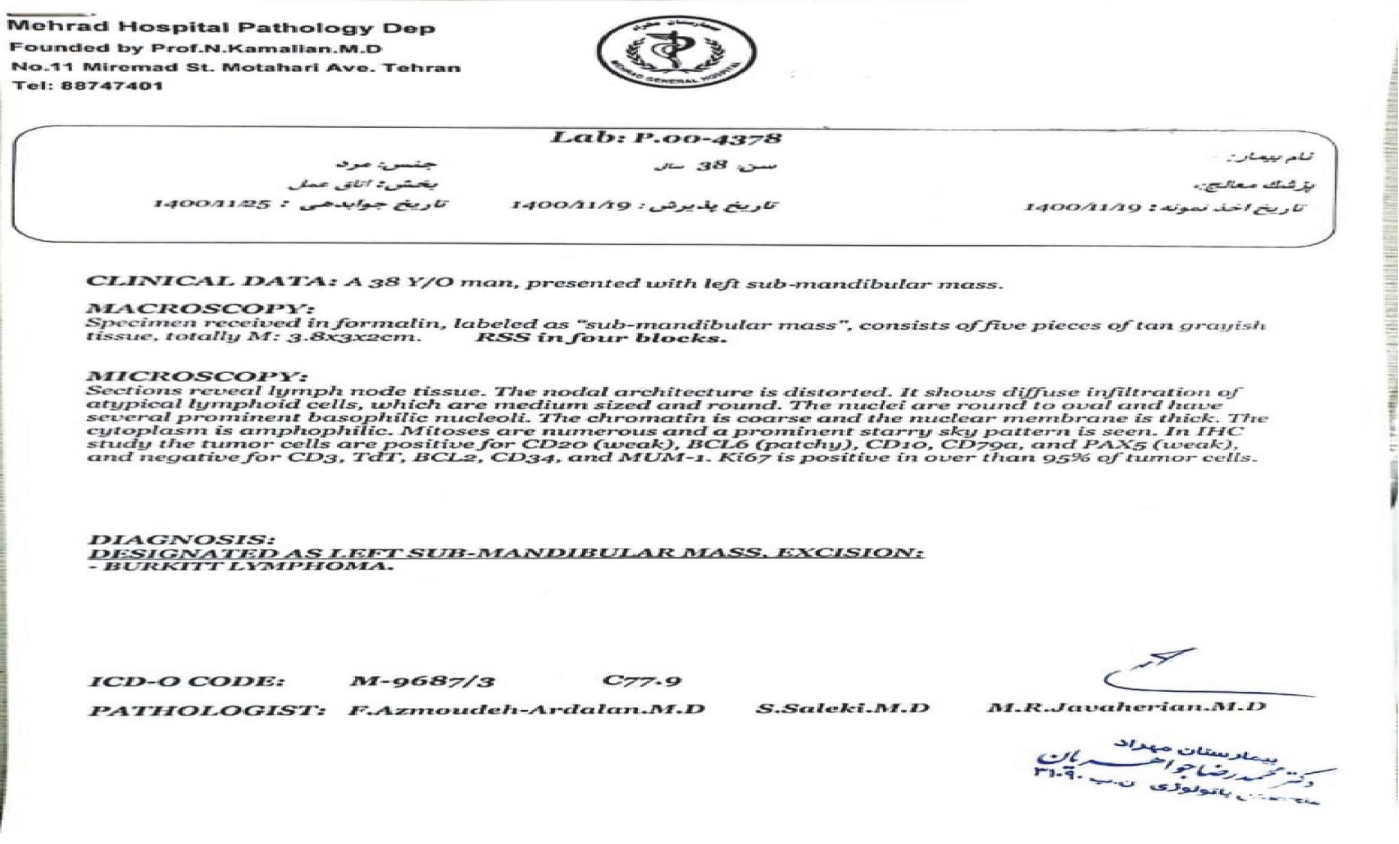
Histological examination.

**Figure 4:**
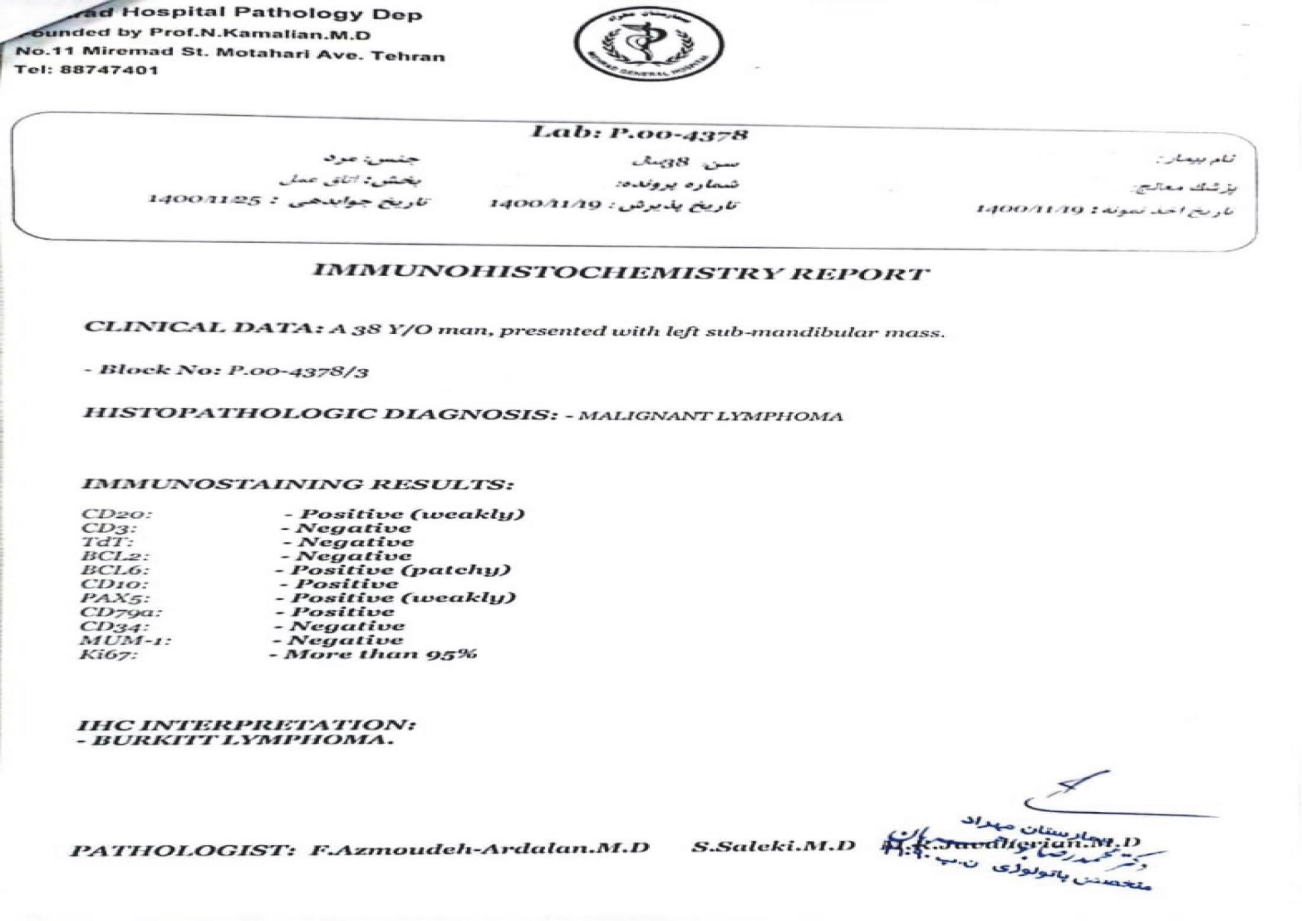
Immunohistochemistry report.

The patient was referred to an oncologist, who recommended an excisional biopsy for further evaluation. The submandibular mass was surgically removed and sent for histopathological and IHC analysis. Histological examination revealed lymph node tissue with distorted architecture, characterized by diffuse infiltration of atypical medium‐sized lymphoid cells. These cells had round to oval nuclei with coarse chromatin, thick nuclear membranes, and prominent basophilic nucleoli. The cytoplasm was amphophilic, mitotic activity was abundant, and a classic “starry sky” pattern was observed (Figure 3 and File S1). The IHC analysis showed that the tumor cells were positive for CD20 (weak), BCL6 (patchy), CD10, CD79a, and PAX5 (weak), while negative for CD3, TdT, BCL2, CD34, and MUM‐1. Additionally, the Ki‐67 proliferation index was greater than 95%, confirming the diagnosis of Burkitt’s lymphoma (Figure 4 and File S1).

**Figure 5:**
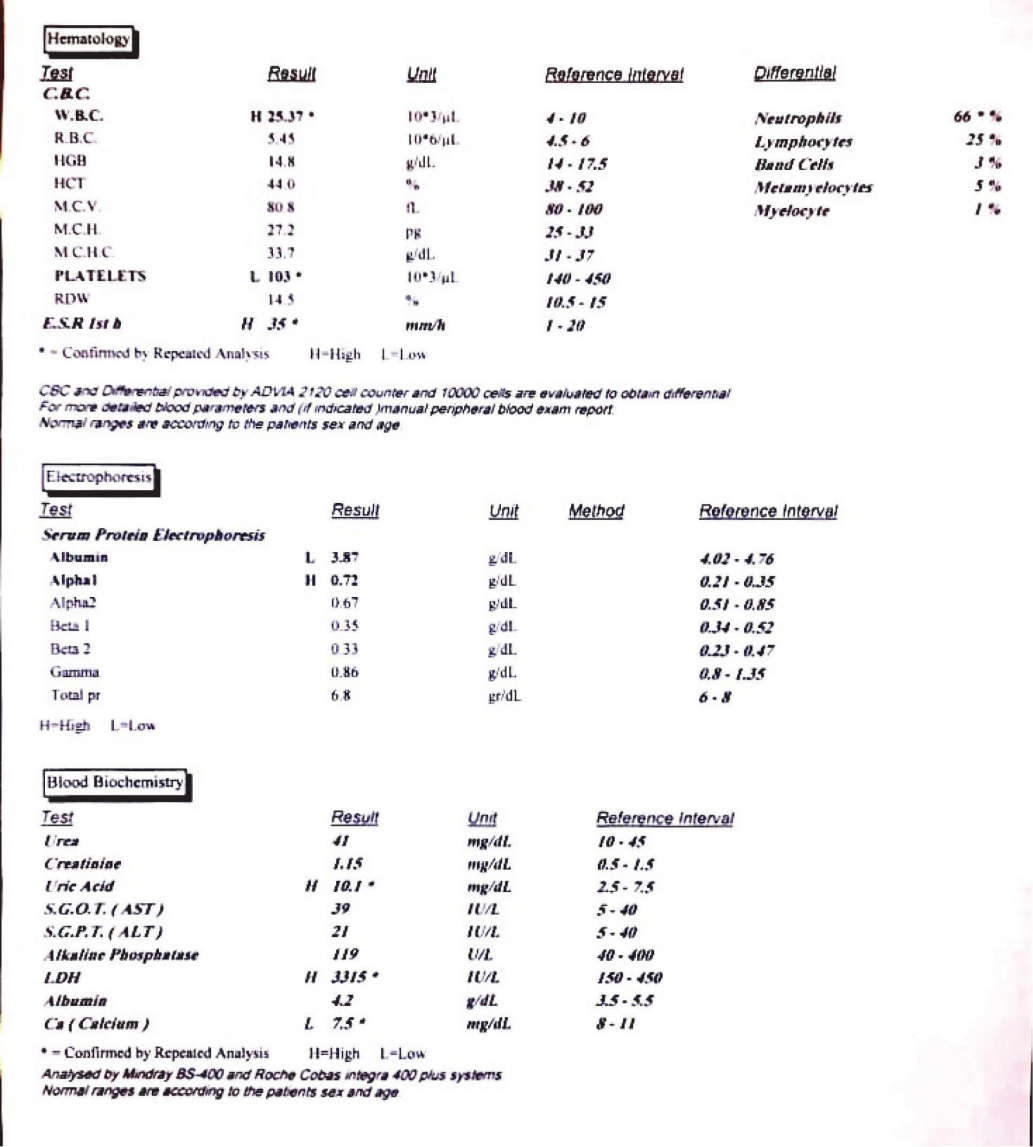
Blood test report.

Simultaneously, routine blood tests indicated abnormalities, including thrombocytopenia (platelets at 103 × 10^9^/L), leukocytosis (white blood cells at 25.37 × 10^9^/L), elevated erythrocyte sedimentation rate (35 mm/h), hypocalcemia (calcium at 7.5 mg/dL), hyperuricemia (uric acid at 10.1 mg/dL), elevated LDH (at 3315 IU/L), and raised C‐reactive protein (27.1 mg/L) (Figure 5).

**Figure 6:**
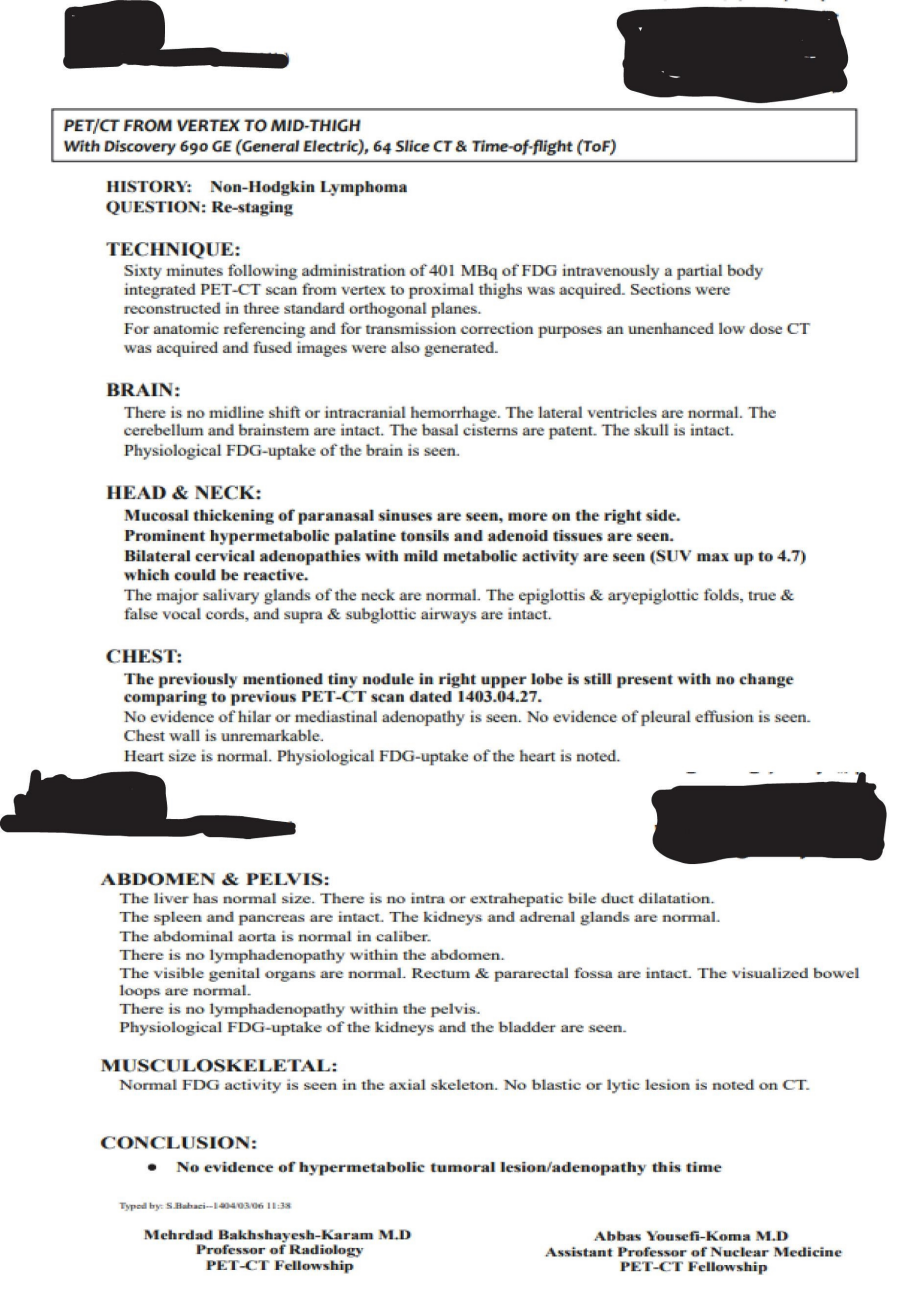
Four‐year follow‐up PET scan report.

**Figure 7:**
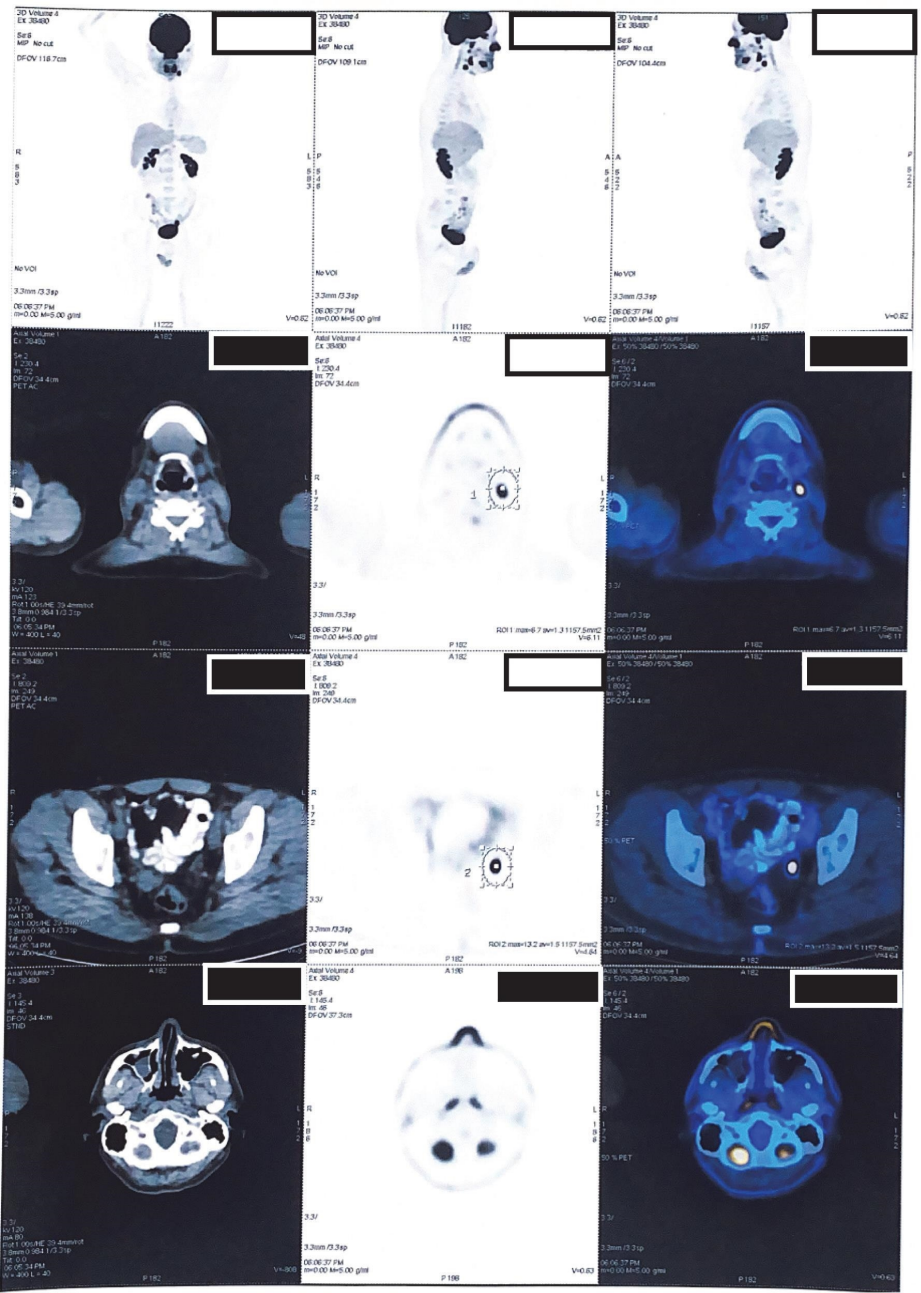
Four‐year follow‐up PET scan showing tiny nodule in right upper lobe with no change comparing to previous scan.

The patient underwent a combined treatment regimen of chemotherapy and radiotherapy. After 3 years of follow‐up, he completed treatment and remains in remission. After 4 years of follow‐up, pet scan revealed tiny nodule in right upper lobe with no change comparing to previous scan (Figures 6 and 7).

## 3. Ethical Consideration

This study was conducted under ethical approval (Code IR.GUMS.REC.1403.264), and the patient obtained informed consent.

## 4. Discussion

Burkitt’s lymphoma is a rare and aggressive type of NHL in adults [11]. While it is most commonly observed in African children, it can also occur in adults from other regions. In Africa, this malignancy accounts for approximately 50% of all pediatric cancers, whereas in different parts of the world, it represents less than 2% of NHL cases [12]. The endemic form of Burkitt’s lymphoma, which typically manifests as a jaw tumor and often spreads to extranodal sites such as the bone marrow and leptomeninges, is not normally seen in adults [13]. An international study investigating Burkitt’s lymphoma in the head and neck region identified the most frequently affected anatomical sites as the cervical lymph nodes (26.92%), both the maxilla and mandible (15.38%), the mandible alone (13.46%), and the maxilla alone (11.54%) [13]. The most prevalent clinical presentation was tumor or swelling (31.25%), followed by cervical lymphadenopathy (10.94%), pain (9.38%), bone destruction (9.38%), and tooth mobility (7.03%) [14].

The rarity of Burkitt’s lymphoma in Iran, combined with its unusual presentation in the oral cavity and the patient’s age, made the diagnosis of this condition unexpected [15]. When Burkitt’s lymphoma involves the lower jaw, the most common clinical features include dental pain and perioral numbness [16]. In the present case, the primary symptoms were dull dental pain and paresthesia on the left side of the mandible, radiating towards the periauricular region. These manifestations were initially attributed to an odontogenic infection, but further investigation revealed Burkitt’s lymphoma as the underlying cause [17].

Parker and Jones [18] described a case of Burkitt’s lymphoma presenting with dental pain and submandibular swelling, which resulted in asymmetry of the neck. In contrast, the present case revealed palpable lymph nodes in the submandibular and jugulodigastric regions without noticeable swelling or neck asymmetry [18]. Additionally, no such signs were observed while the earlier case reported abnormalities at the tooth extraction site and painless lumps on the scalp. The involvement of the mental and inferior alveolar nerves and persistent symptoms following tooth extraction strongly indicated the need to investigate alternative underlying pathologies. Early and accurate diagnosis significantly improves outcomes, as most patients with Burkitt’s lymphoma respond well to appropriate treatment [18].

The findings of Cho et al. [19] describe a presentation in which hypermobility of the maxillary right first molar was the initial clinical sign, followed by the identification of painless gingival and mucosal swelling in the posterior regions of both the maxilla and mandible. In contrast, the patient in our study presented primarily with a complaint of dull pain and paresthesia localized to the left side of the mandible. This discrepancy underscores a significant variability in the initial symptomatic presentation of the condition [19].

Based on the study of Bello‐Castro et al. [10], low hemoglobin levels, elevated levels of β2‐microglobulin and LDH, and being in more advanced stages (III or IV) are associated with poorer prognosis. In this case, while the levels of hemoglobin and β2‐microglobulin were within the normal range, the patient presented with elevated LDH levels. This discrepancy indicates a potential risk factor that could influence the overall prognosis despite the other regular markers.

In alignment with the most recent 2025 National Comprehensive Cancer Network (NCCN) guidelines [20], the therapeutic approach to Burkitt lymphoma requires age‐ and risk‐stratified multiagent chemotherapy, preferably administered at a center with expertise in this disease. For patients these patients, preferred regimens include dose‐adjusted EPOCH plus rituximab, CODOX‐M ± rituximab, or hyperCVAD ± rituximab, all of which incorporate CNS prophylaxis with intrathecal therapy—a critical consideration given the orofacial presentation and its potential for CNS involvement. The guidelines further emphasize that CHOP is inadequate, mandate prophylaxis for tumor lysis syndrome, and recommend a structured follow‐up schedule of every 2–3 months for the first year following complete response.

## 5. Conclusion

This case highlights the importance of thoroughly evaluating paresthesia in the lower jaw and periauricular region and other concerning symptoms in primary care settings. When initial findings and treatments fail to resolve the issue, prompt referral to a specialist is crucial. Advanced diagnostic approaches, including cone beam CT (CBCT), US, and the excision of abnormal soft tissues for histopathological and IHC analysis, play a pivotal role in identifying the underlying condition.

## Author Contributions

**Mona Shameli:** conceptualization (formulation of research goals and aims), methodology, writing – original draft (drafting and preparation of the full manuscript text), writing – review and editing (critical revision and refinement of the manuscript). **Pedram Hajibagheri**: methodology, writing – review and editing (critical review and language editing), supervision (oversight and guidance throughout the study). **Amirreza Hendi**: supervision (overall oversight and academic guidance), writing – review and editing (final review and approval of the manuscript). **Mahsa Koochaki**: writing – original draft (drafting and preparation of the full manuscript text), supervision (overall oversight and academic guidance), writing – review and editing (final review and approval of the manuscript).

## Funding

No funding was received for this manuscript.

## Disclosure

After using Deepseek, the authors reviewed and edited the content as necessary, taking full responsibility for the final publication.

## Consent

The patient provided a signed consent form, allowing the authors to publish his treatment details.

## Conflicts of Interest

The authors declare no conflicts of interest.

## Supporting Information

Additional supporting information can be found online in the Supporting Information section.

## Supporting information


**Supporting Information** File S1. Histopathological sections with magnification details.

## Data Availability

The data that support the findings of this study are available upon request from the corresponding author.
