# Supplementary material for: Novel Case Report of a Rare Symptomatic Orofacial Burkitt’s Lymphoma
Source: Case Rep Dent. 2026 Apr 29;2026:9669783. doi: 10.1155/crid/9669783 (PMC13127233; doi:10.1155/crid/9669783)
Supplement: Supplementary file 1 — Supporting Information File S1. Histopathological sections with magnification details. [file CRID-2026-9669783-s001.docx]

| CD3 negetive | 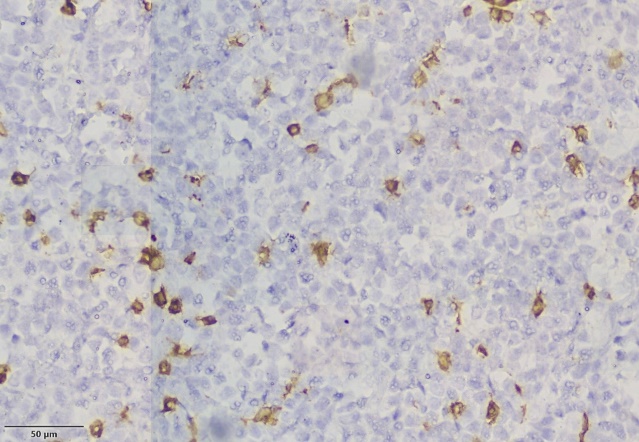 |
| --- | --- |
| H&E | 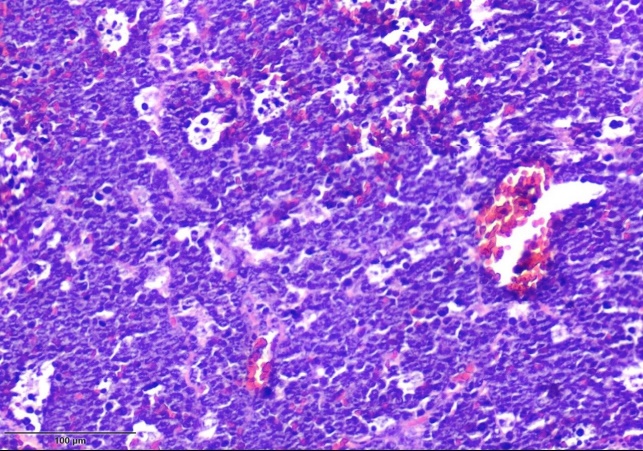 |
| CD79a-positive | 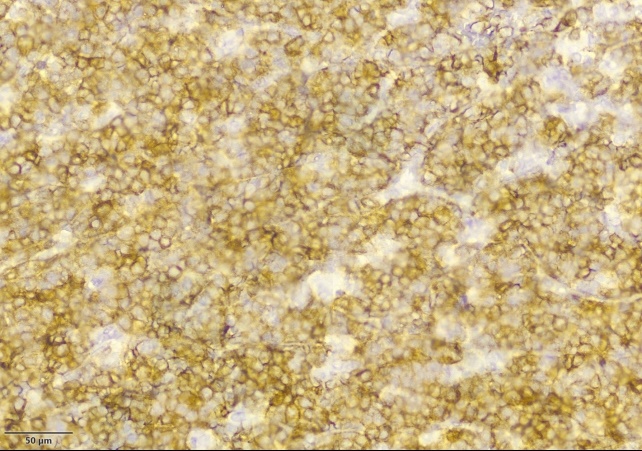 |
| BCL6 Positive in most tumor cell | 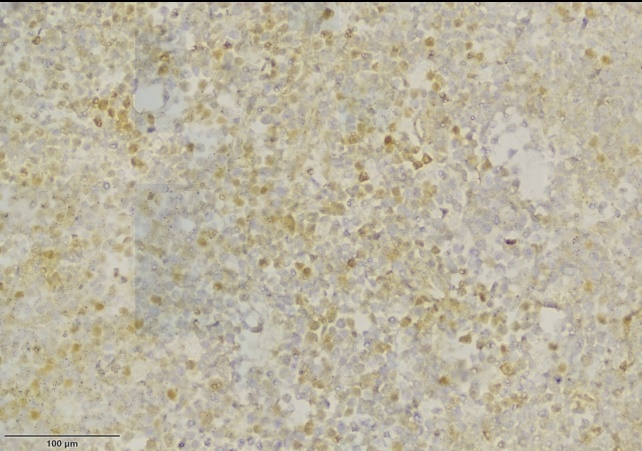 |
| 12MUM-1 negetive | 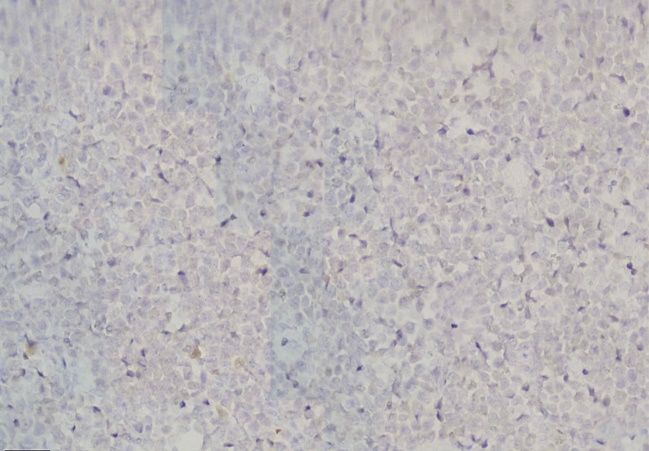 |
| CD10 | 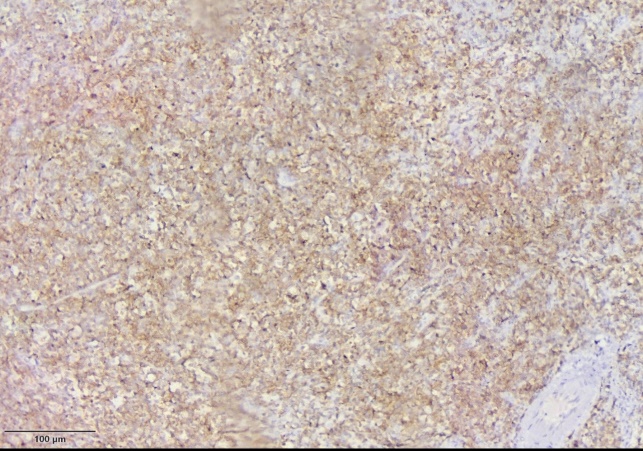 |
| Hematoxilin & Eosin | 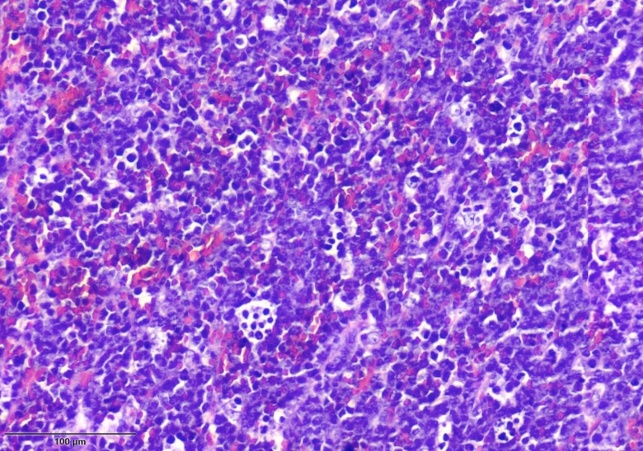 |
| Low-power . | 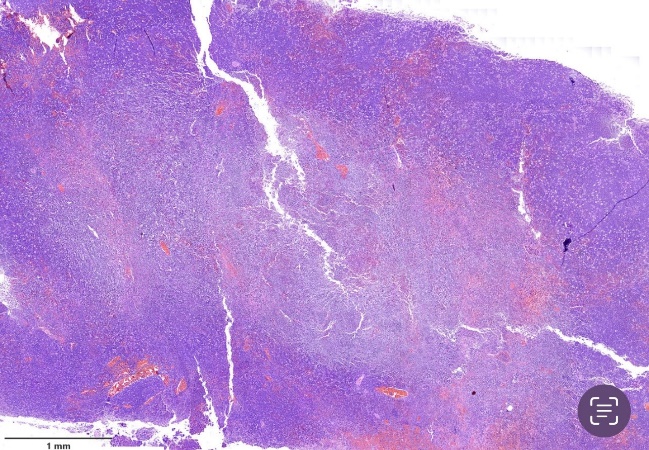 |
| CD3 negetive | 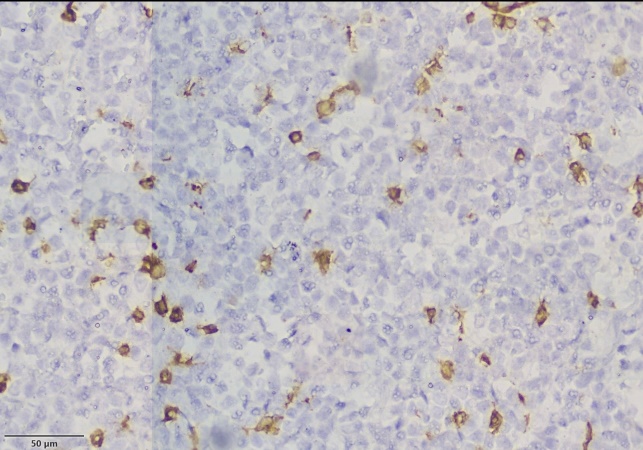 |
| BCL2 negetive | 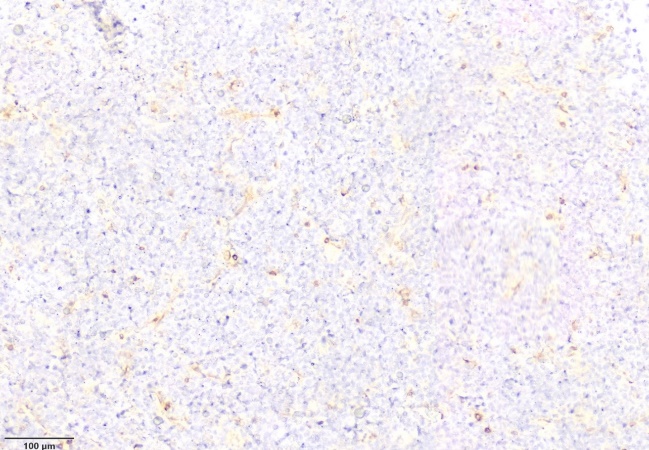 |
| H&E Sttary sky appearance | 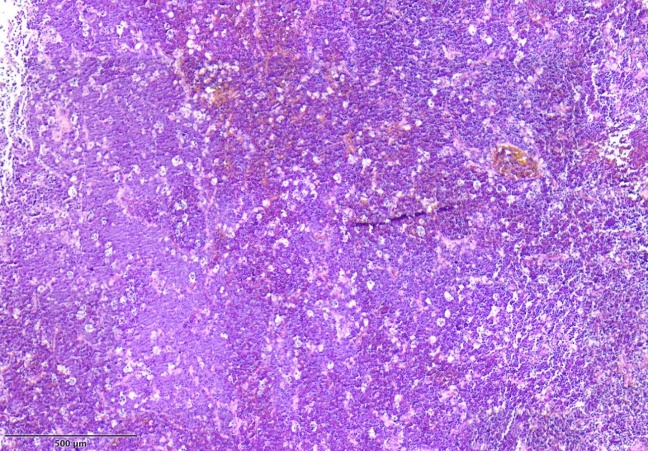 |
| H&E starry sky | 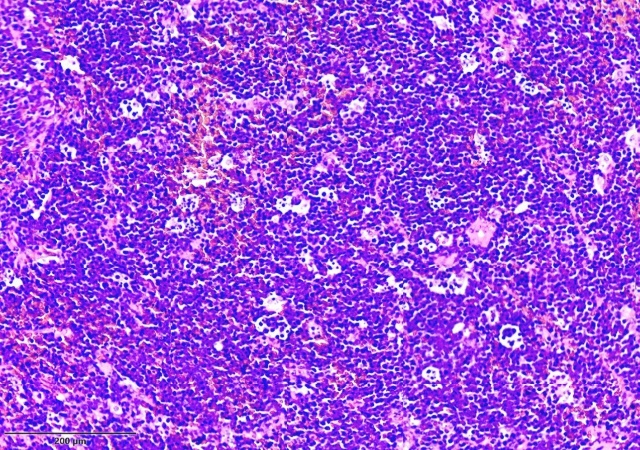 |
| Ki 67high -proliferation index | 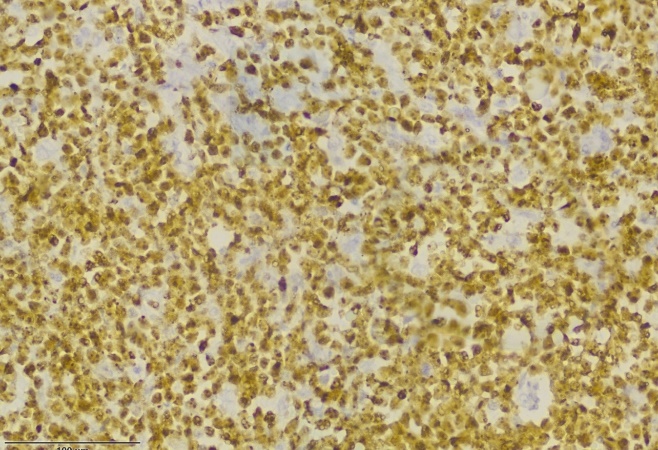 |
